# Differential effects of depot medroxyprogesterone acetate administration on vaginal microbiome in Hispanic White and Black women

**DOI:** 10.1080/22221751.2018.1563458

**Published:** 2019-01-21

**Authors:** Liying Yang, Yuhan Hao, Jiyuan Hu, Dervla Kelly, Huilin Li, Stuart Brown, Carley Tasker, Natalie E. Roche, Theresa L. Chang, Zhiheng Pei

**Affiliations:** a Department of Pathology, New York University School of Medicine, New York, NY, USA; b Department of Medicine, New York University School of Medicine, New York, NY, USA; c Applied Bioinformatics Laboratories, New York University School of Medicine, New York, NY, USA; d Department of Biology, Center for Genomics and Systems Biology, New York University, New York, NY, USA; e Department of Population Health, New York University School of Medicine, New York, NY, USA; f Department of Environmental Medicine, New York University School of Medicine, New York, NY, USA; g Department of Microbiology, Biochemistry and Molecular Genetics, Rutgers, The State University of New Jersey, New Jersey Medical School, Newark, NJ, USA; h Department of Obstetrics, Gynecology & Women’s Health, Rutgers, the State University of New Jersey, New Jersey Medical School, Newark, NJ, USA; i Public Health Research Institute, Rutgers, The State University of New Jersey, New Jersey Medical School, Newark, NJ, USA; j Department of Veterans Affairs New York Harbor Healthcare System, New York, NY, USA

**Keywords:** Vaginal microbiome, depot medroxyprogesterone acetate, *Lactobacillus*, bacterial vaginosis-associated bacteria, network, HIV acquisition

## Abstract

The use of depot medroxyprogesterone acetate (DMPA), a 3-monthly injectable hormonal contraceptive, is associated with an increased risk of HIV acquisition possibly through alteration of the vaginal microbiome. In this longitudinal interventional study, we investigated the impact of DMPA administration on the vaginal microbiome in Hispanic White and Black women at the baseline (visit 1), 1 month (visit 2), and 3 months (visit 3) following DMPA treatment by using 16S rRNA gene sequencing. No significant changes in the vaginal microbiome were observed after DMPA treatment when Hispanic White and Black women were analysed as a combined group. However, DMPA treatment enriched total vaginosis-associated bacteria (VNAB) and *Prevotella* at visit 2, and simplified the correlational network in the vaginal microbiome in Black women, while increasing the network size in Hispanic White women. The microbiome in Black women became more diversified and contained more VNAB than Hispanic White women after DMPA treatment. While the *Firmicutes* to *Bacteroidetes* (F/B) ratio and *Lactobacillus* to *Prevotella* (L/P) ratio were comparable between Black and Hispanic White women at visit 1, both ratios were lower in Black women than in Hispanic White women at visit 2. In conclusion, DMPA treatment altered the community network and enriched VNAB in Black women but not in Hispanic White women. The *Lactobacillus* deficiency and enrichment of VNAB may contribute to the increased risk of HIV acquisition in Black women. Future studies on the impact of racial differences on the risk of HIV acquisition will offer insights into developing effective strategies for HIV prevention.

**Abbreviations:** DMPA: depot medroxyprogesterone acetate; PCR: polymerase chain reaction; OTU: operational taxonomic unit; STI: sexually transmitted infections; VNAB: vaginosis-associated bacteria

## Introduction

Depo-Provera (depot medroxyprogesterone acetate, DMPA), an injectable form of hormonal contraceptive given every 3 months, is associated with an increased risk of HIV acquisition and transmission [1–3]. The contribution of DMPA to an increase in HIV transmission is likely to be multifaceted [4]. Studies on biological mechanisms of heightened HIV transmission by DMPA indicate that DMPA may increase HIV susceptibility by alteration of epithelial permeability [5,6], immune response [4,7,8], and vaginal microbiome [9], although the dynamics of vaginal microbiome in response to DMPA are less defined.

The vaginal microbiome comprises a dynamic ecosystem with important host defense capabilities integral to reproductive health [10,11]. Epidemiological evidence suggests that the normal vaginal microbiome may play a critical role in reducing the risk of sexually transmitted infections (STIs), including HIV-1 [10,12]. The vaginal microbiome has been classified into several types based on specific *Lactobacillus* species and the abundance of *Lactobacillus* [11]. Studies on microbiome dynamics in the reproductive cycle in different populations indicate that the type of species present in the vaginal microbiome remain constant throughout the cycle, although their abundance varies in different community state types (CSTs) [10] and different racial groups [13]. In the vagina of healthy women, specific *Lactobacillus* species can directly preclude exogenous bacteria from stable colonization, inhibit overgrowth of harmful endogenous bacteria by competing for essential nutrients and mucosal attachment sites and suppress growth through the secretion of bacteriocin, lactic acid, and hydrogen peroxide [14–17]. The dominance of *Lactobacillus* is associated with a healthy vaginal environment [18]. Depletion of vaginal *Lactobacillus* is associated with an increased risk of acquiring HIV and STIs [19]. For example, bacterial vaginosis (BV), a polymicrobial syndrome resulting from the loss of *Lactobacillus* species and the enrichment of anaerobic bacteria, is associated with a heightened inflammatory state [20] and an increased risk of HIV acquisition [21–23]. However, 40% of Black and Hispanic women have vaginal microbiota that are diverse (CST IV) and non-*Lactobacillus* dominant, but do not have clinical symptoms of disease [11]. This suggests that certain women can have diverse microbiota and be “healthy”.

The effect of DMPA on the vaginal microbiome in women has been studied to a limited extent. Cultivation-based longitudinal studies with subjects shows that there is a significant decrease in vaginal H_2_O_2_-positive *Lactobacillus* 6 and 12 months after DMPA injection, although there is no change in the abundance of total *Lactobacillus* [24,25]. The analysis of specific *Lactobacillus* species and *Gardnerella vaginalis* longitudinally by quantitative PCR in 15 Kenyan women with one-year DMPA treatment indicates decrease in vaginal *G. vaginalis* and total bacteria (16S rRNA gene) in response to DMPA [9]. There is no change in *L. iners* in response to DMPA but *L. crispatus* is detected in 2 of the 15 women before and 9 of 15 women after DMPA treatment but change in abundance was not reported [9]. However, a similar longitudinal study in Zimbabwean women shows a decrease in *L. iners* but no changes in beneficial *lactobacillus* species (*L. crispatus, L. gasseri, L. jensenii*) in response to DMPA [26]. A cross-sectional study by using 16S rRNA gene sequencing shows that DMPA users have less abundant vaginosis-associated bacteria (VNAB) compared to condom users, although DMPA use is associated with higher levels of *L. iners* and vaginal dysbiosis including *Atopobium vaginae, Dialister microaerophilus, Prevotella bivia, Prevotella amnii and Anerococcus christensenii* [27]. Overall, the changes in the abundance of specific *Lactobacillus* species by DMPA were inconsistent. Interestingly, a cross-sectional study using tandem-mass spectrometry [28] and a longitudinal study by phylogenetic microarrays [29] did not find significant alteration of the vaginal microbiome by DMPA in Kenyan women and female sex workers in Rwanda, respectively.

Culture-dependent methods of microbial identification are the gold standard for diagnosis of infections but are extremely biased towards microbes that can readily be cultured in laboratory settings. Culture-dependent studies often select only one or a few species rather than define the entire microbial community because the requirement of specific culture conditions differs greatly among the community members. Furthermore, these studies report results as culture-positive or negative rather than quantifying taxon abundance because quantification is difficult for bacteria with different growth rates and nutritional requirements. Thus, full bacterial diversity cannot be explored in a complex microbial community with culture-dependent methods. PCR-based methods are a type of culture-independent methods that can be quantitative but are limited to selected species. These limitations are exemplified by the number of attempts to date studying the DMPA effect on the vaginal microbiome with noncomparable results. To overcome these drawbacks, broad range culture-independent methods have been developed [30,31]. The most commonly used culture-independent method relies on amplification and analysis of the 16S rRNA genes in a microbiome. 16S rRNA genes are widely used for documentation of the evolutionary history and taxonomic assignment of individual organisms [32] because they have highly conserved regions for construction of universal primers and highly variable regions for identification of individual species. When coupled with next generation sequencing, 16S rRNA gene survey can recover nearly all bacteria and have revealed thousands of species of bacteria in microbiomes previously thought to be dominated by a few cultivable species.

Understanding alteration of vaginal microbiome by DMPA has potential implications in women who are at a risk of acquiring HIV and other STIs. Given the complexity of vaginal bacterial community structures in individuals, we designed a longitudinal study to detect vaginal microbiome changes in individual women in response to DMPA using high throughput 16S rRNA gene sequencing. We analysed vaginal microbiome in samples from women before (baseline, visit 1), 1 month (visit 2), and 3 months following (visit 3) DMPA injection. Because plasma DMPA concentrations are known to decrease over time and return to near baseline 3 months after infection [33,34], these time points will allow us to detect microbiome changes in response to DMPA (visit 2) as well as to address the sustained effect of DMPA on microbiome after the decline of DMPA concentration (visit 3). Our results indicated that DMPA changed the vaginal microbiome and this change was affected by race and duration of treatment.

## Results

A total of 25 healthy subjects (9 Hispanic White and 16 Black women) meeting the eligibility criteria were enrolled during May 2015 to May 2016. Among them, 16 participants returned for all visits, 6 participants returned only for visit 2, and 3 participants returned only for visit 3 (Supplementary Table S1). None of the subjects used any form of hormonal contraception including DMPA for up to 10 months before the time of recruitment. Bacterial 16S rRNA genes were sequenced using Miseq. On average, each sample yielded 20,633.65 (SD ± 13,154.94, range: 4,892–98,000) high quality reads. Overall, the reads were agglomerated to 7 phyla and 29 genera, after removal of non-bacterial reads, unclassifiable taxa, and low abundance taxa (<0.1%) (Supplementary Table S2).

## DMPA use did not globally affect the α- and β-diversities of the vaginal microbiome

In an analysis of the effect of DMPA use on α-diversity (how many bacterial species are in the community, and how equally distributed those bacterial organism counts are among each other in a sample), we found that α-diversity of the vaginal microbiome in all women did not attain statistical significance among visits 1, 2, and 3 and between any two of the three visits by either observed OTU, Chao1-predicted OTUs, or Shannon diversity index (Supplementary Table S3). By race, neither the Hispanic White (White) nor Black women differed significantly among the three visits or between any two of the three visits (Supplementary Table S3).

In an analysis of the effect of DMPA use on β-diversity (how many bacterial species are unique in the communities compared, weighted or unweighted by abundance), we found that β-diversity of the vaginal microbiomes were not statistically significant among the three visits or between any pairs of the visits by either weighted or unweighted UniFrac (Supplementary Table S4). By race, neither the White nor Black women differed significantly among the three visits or between any pairs of the three visits (Supplementary Table S4).

## DMPA use enriched vaginosis-associated bacteria in the vaginal microbiome in Black women

Using the Wilcoxon signed-rank test for paired samples within each woman at different visits, we compared the vaginal microbiomes pre- and post-DMPA treatment by taxonomic abundance. In all women combined, DMPA use transiently enriched *Prevotella* (*p* = 0.0391, *q *= 0.3777), *Fusobacterium* (*p* = 0.0251, *q *= 0.3645), and *Anaerococcus* (*p* = 0.0229, *q *= 0.3645) at visit 2 compared with the baseline at visit 1 (Supplementary Figure 1a). However, the differences were no longer significant after false discovery correction. Similarly, *Atopobium* (*p* = 0.0182, *q *= 0.5292) and *Flexispira* (*p* = 0.0443, *q *= 0.6425) differed significantly between visits 2 and 3 before false discovery correction (Supplementary Figure 1b). No difference in the abundance of any bacterial genera was found between visits 1 and 3. DMPA treatment did not significantly change the total VNAB abundance.

**Figure 1.**
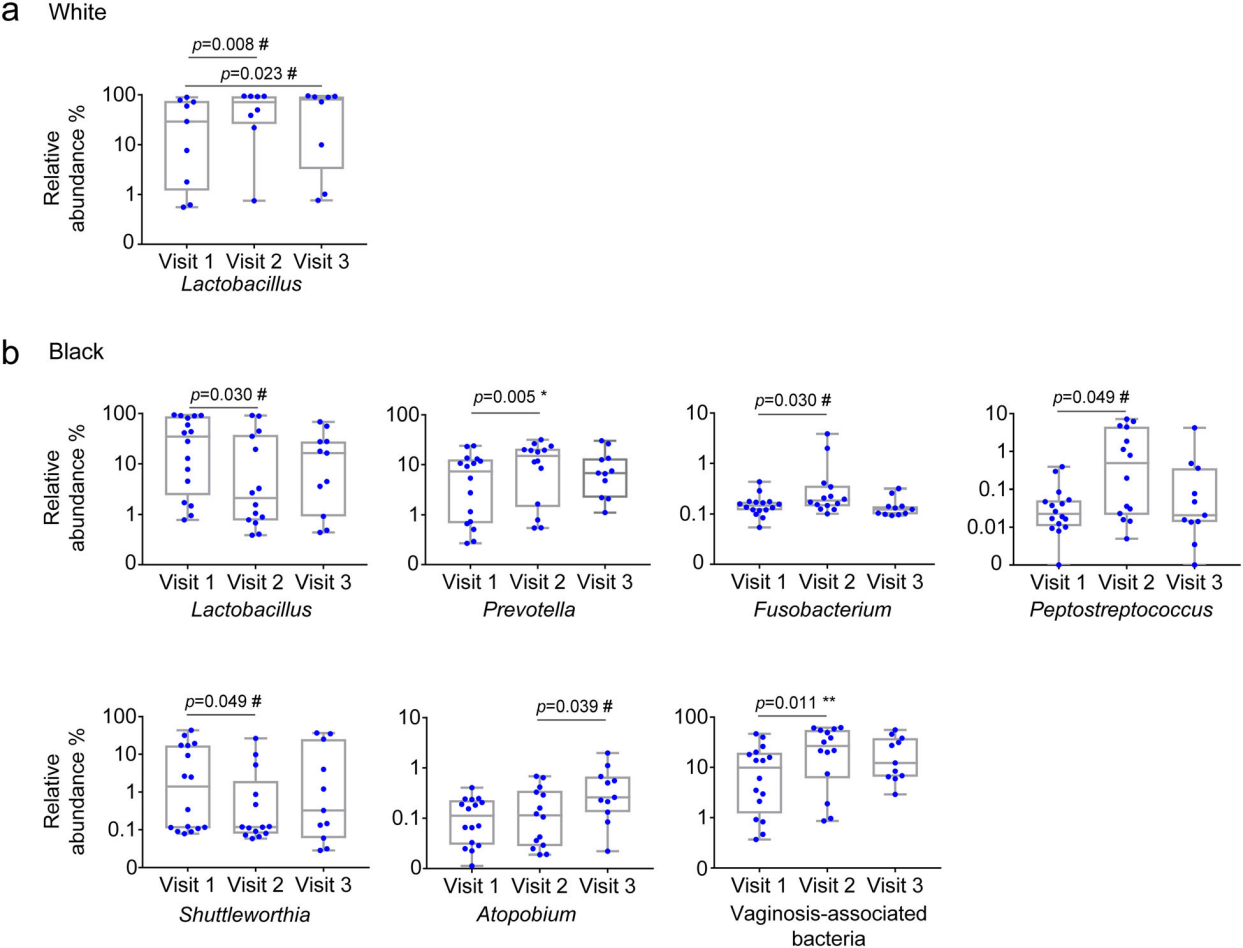
Differential genera between naive and DMPA-treated vaginal microbiomes in Hispanic White and Black women. Microbiome of vaginal swabs from the participants with pre- and post-DMPA administration was analysed. Difference in the relative abundance of genera was evaluated between two of the three visits using the Wilcoxon signed rank test for paired samples for White (a) and Black (b) women. A change in the microbial composition was considered as significant if *p* < 0.05 and FDR adjusted *p* < 0.20 (#>0.2, * ≤ 0.2, ** ≤ 0.1, *** ≤ 0.05).

We further performed taxonomic analysis by race. In White women, DMPA use enriched *Lactobacillus* before false discovery correction at visit 2 (*p* = 0.0078, *q *= 0.2266) and at visit 3 (*p* = 0.0234, *q *= 0.6797) compared with the baseline (Figure 1(a)). None of genera showed statistical differences between visits 2 and 3.

In Black women, there was an increase in the VNAB *Prevotella* (*p* = 0.0052, *q *= 0.1522) at visit 2 compared to the baseline, and the difference remained significant after false discovery correction (Figure 1(b)). There was an increase in *Fusobacterium* (*p* = 0.0295, *q *= 0.2856) and *Peptostreptococcus* (0.0494, *q *= 0.2867) and a decrease in *Shuttleworthia* (*p* = 0.0494, *q *= 0.2867) at visit 2 compared with the baseline visit 1 before false discovery correction (Figure 1(b)). Furthermore, there was a decrease in *Lactobacillus* (*p* = 0.0295, *q *= 0.2856) at visit 2 compared with visit 1 before false discovery correction (Figure 1(b)). *Atopobium* (*p* = 0.0391, *q *= 0.7174) was significantly more abundant at visit 3 compared with visit 2 before false discovery correction (Figure 1(b)). No difference in any genus was found between visits 1 and 3.

We analysed VNAB species in our samples. Of the previously defined nine genera associated with BV [35], *Mobiluncus* and *Leptotrichia* were filtered out from our dataset because their relative abundances were below our inclusion criterion (0.1%). The remaining seven VNAB genera included *Prevotella, Sneathia, Gardnerella, Atopobium, Clostridium, Peptostreptococcus* and *Mycoplasma* (Table 1). By prevalence, nearly all genera were detected in the large majority of the samples either before or after DMPA treatment. Besides the aforementioned increase in *Prevotella* abundance, DMPA also significantly increased the total VNAB abundance at visit 2 in Black women (median combined abundance 9.84, vs. 26.73%, *p* = 0.011, *q *= 0.066) (Figure 1(b)) (Table 2). We analysed the three bacterial pathogens to species level for STIs including *Neisseria gonorrhoeae, Chlamydia trachomatis,* and *Treponema pallidum,* and did not detect these species in any of the participants at any visits.

**Table 1.** Prevalence and abundance of vaginosis-associated bacteria and Lactobacillus in the vaginal microbiome before and after DMPA treatment.

	Prevalence % (Median abundance %)					
Race	All Hispanic White participants			All Black participants		
Clinical visit	Visit 1	Visit 2	Visit 3	Visit 1	Visit 2	Visit 3
*Prevotella*	100 (0.65)	100 (0.37)	100 (0.86)	100 (7.32)	10 (14.96)	100 (6.77)
*Sneathia*	100 (0.08)	100 (0.05)	100 (0.07)	100 (0.65)	100 (5.15)	100 (0.44)
*Gardnerella*	100 (0.15)	87.5 (0.05)	100 (0.11)	100 (0.26)	100 (0.22)	100 (0.29)
*Atopobium*	100 (0.05)	87.5 (0.02)	87.5 (0.03)	100 (0.11)	100 (0.11)	100 (0.26)
*Clostridium*	75.0 (0.02)	100 (0.02)	100 (0.03)	100 (0.27)	100 (0.36)	100 (0.49)
*Peptostreptococcus*	87.5 (0.05)	100 (0.04)	87.5 (0.03)	93.8 (0.02)	100 (0.49)	90.9 (0.02)
*Mycoplasma*	62.5 (0.01)	75.0 (0.01)	100 (0.02)	93.8 (0.02)	87.5 (0.03)	100 (0.01)
All VNAB	100 (2.08)	100 (1.11)	100 (1.99)	100 (9.84)	100 (26.73)	100 (12.25)
*Lactobacillus*	100 (29.02)	100 (70.87)	100 (80.73)	100 (34.88)	100 (2.13)	100 (16.32)

Note: The definition of vaginosis-associated bacteria was as previously described [48]. *Mobiluncus* and *Leptotrichia* were not included in taxonomic analysis because their abundances were below our inclusion criterion (0.1%).

**Table 2.** Paired comparison of total vaginosis-associated bacteria before and after DMPA treatment.

Race	Comparison	Number of pairs	Median abundance %			*p-*value*	*q*-value**
			Visit 1	Visit 2	Visit 3		
Hispanic White	Visit 1 vs. 2	8	2.00	1.11		0.484	0.713
	Visit 1 vs. 3	8	4.64		1.99	0.208	0.416
	Visit 2 vs. 3	7		1.63	0.70	0.886	0.886
Black	Visit 1 vs. 2	14	4.77	20.73		**0**.**011**	**0**.**066**
	Visit 1 vs. 3	11	6.03		12.25	0.128	0.384
	Visit 2 vs. 3	9		31.83	12.25	0.594	0.713

*Wilcoxon signed-rank test.

**FDR-corrected *p*-value by Benjamin and Hochberg method [39].

## The vaginal microbiomes differed significantly between White and Black women before and after DMPA treatment

Because of the racial differences in response to DMPA, we compared the vaginal microbiome between White and Black women at baseline and after DMPA use. For α-diversity (Supplementary Figure S2), the microbiomes of White and Black women did not differ significantly at baseline in observed OTUs, Chao1-predicted OTUs, and Shannon index. After DMPA use, the microbiome of Black women became significantly more diversified than that of the White women in observed OTUs and Shannon index at visit 2 and in Shannon index at visit 3.

**Figure 2.**
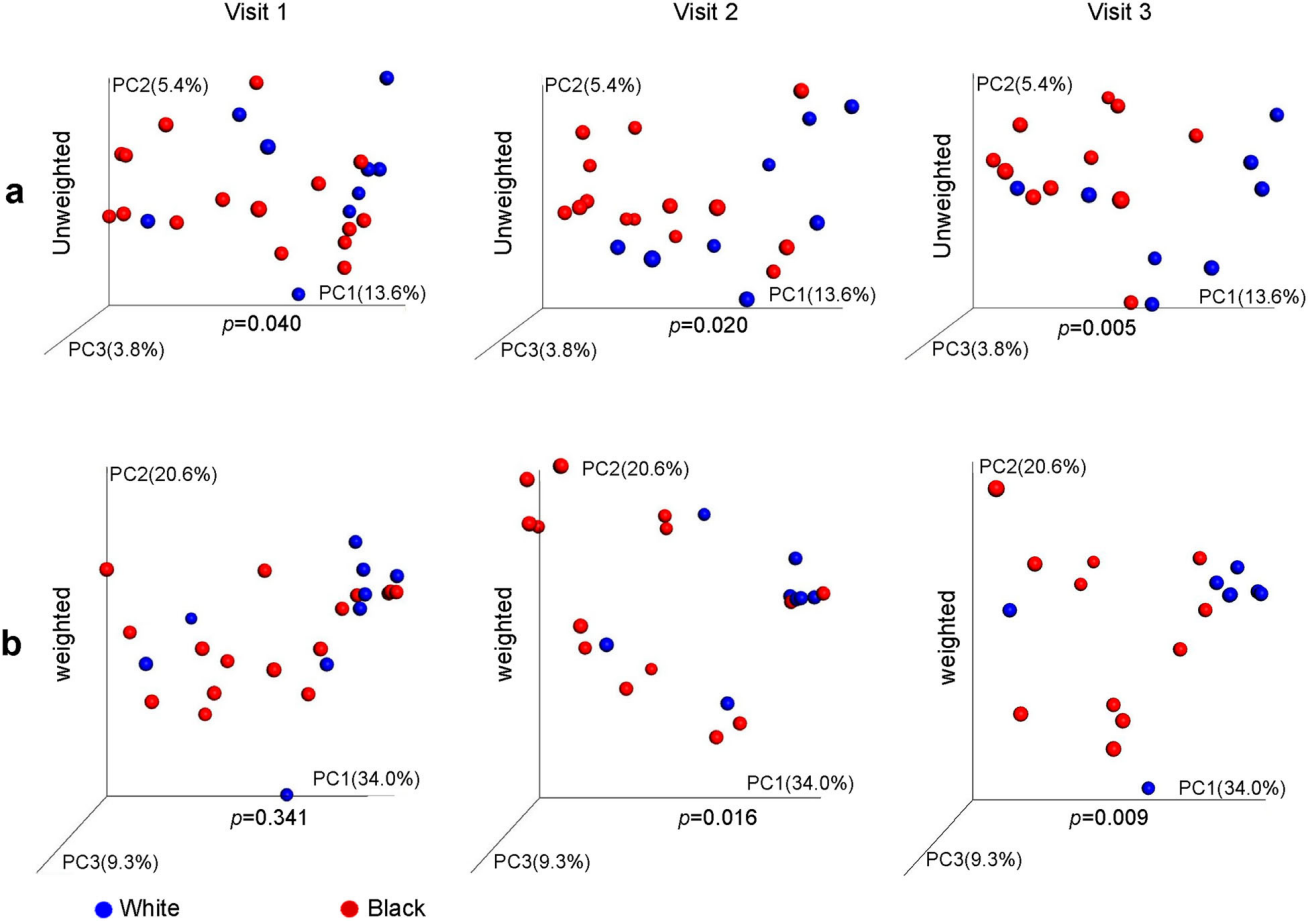
Difference of vaginal microbiome in β-diversity between Hispanic White and Black women before and after DMPA treatment. Vaginal microbiome from the participants with pre- and post-DMPA administration was analysed. β-diversity between White and Black women was analysed by PCoA) using either unweighted (a) or weighted UniFrac distance metrics with *p-*values calculated by microbiome regression-based kernel association test (MiKART).

The β-diversity of microbiomes differed significantly between White and Black women by unweighted UniFrac at the baseline and by either unweighted or weighted UniFrac distance at visits 2 and 3 (Figure 2(a–b)).

**Figure 3.**
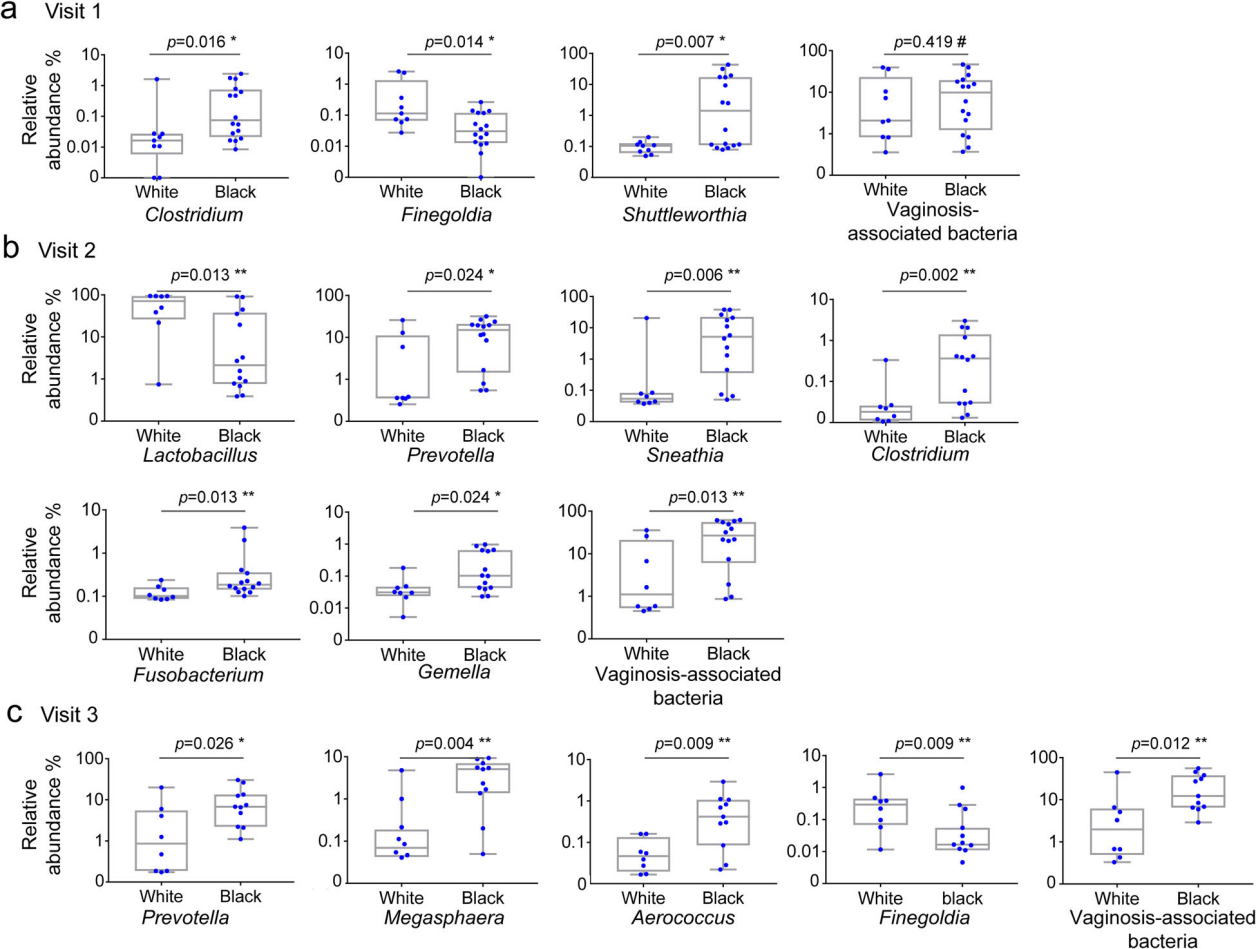
Taxonomic difference of the vaginal microbiome between Hispanic White and Black women before and after DMPA treatment. Vaginal microbiome from the participants with pre- and post-DMPA administration was analysed. Difference in the relative abundance of genera was evaluated between White and Black women using Wilcoxon rank-sum test for unpaired samples at visit 1 (a), visit 2 (b) and visit 3 (c). A change in the microbial composition was considered as significant if *p* < 0.05 and FDR adjusted *p* < 0.20 (#>0.2, * ≤ 0.2, ** ≤ 0.1, *** ≤ 0.05).

Taxonomic analysis revealed that at the baseline, Black women harboured more *Clostridium* (0.0161, *q *= 0.1557), *Finegoldia* (*p* = 0.01389, *q *= 0.1557), and *Shuttleworthia* (*p* = 0.0065, *q *= 0.1557), than White women (Figure 3(a)) (Table 1)*.* At visit 2, five genera including *Prevotella* (*p* = 0.0240, *q =* 0.1161), *Clostridium* (*p* = 0.002, *q =* 0.0629), *Sneathia* (*p* = 0.0064, *q =* 0.0923), *Fusobacterium* (*p* = 0.0128, *q =* 0.0931), and *Gemella* (*p* = 0.0240, *q =* 0.1161) were significantly more abundant, while *Lactobacillus* was less abundant (*p* = 0.0128, *q =* 0.0931) in Black women than in White women (Figure 3(b)). At visit 3, *Prevotella* (*p* = 0.0259, *q =* 0.1880), *Megasphaera* (*p* = 0.0036, *q =* 0.0880), *Finegoldia* (*p* = 0.0091, *q =* 0.0880), and *Aerococcus* (*p* = 0.0091, *q =* 0.0880) were significantly more abundant in Black women than in White women. In addition, total VNAB abundance did not differ significantly between White and Black women at the baseline, but became more abundant in Black women than White women at visit 2 (*p* = 0.013, *q =* 0.078) and at visit 3 (*p* = 0.012, *q =* 0.090) (Figure 3(b–c); Table 1).

## DMPA treatment altered the correlational network of the vaginal microbiome

Besides α- and β-diversities, a microbiome can also be characterized by the degree of interaction among its community members using a correlational network analysis. In the network, a connection is the relationship or correlation between two bacterial variables in which both variables move in tandem or in inversely. A positive connection exists when one bacterium decreases as the other bacterium decreases, or one variable increases while the other increases. Negative connection exists when one bacterium decreases while the other bacterium increases.

**Figure 4.**
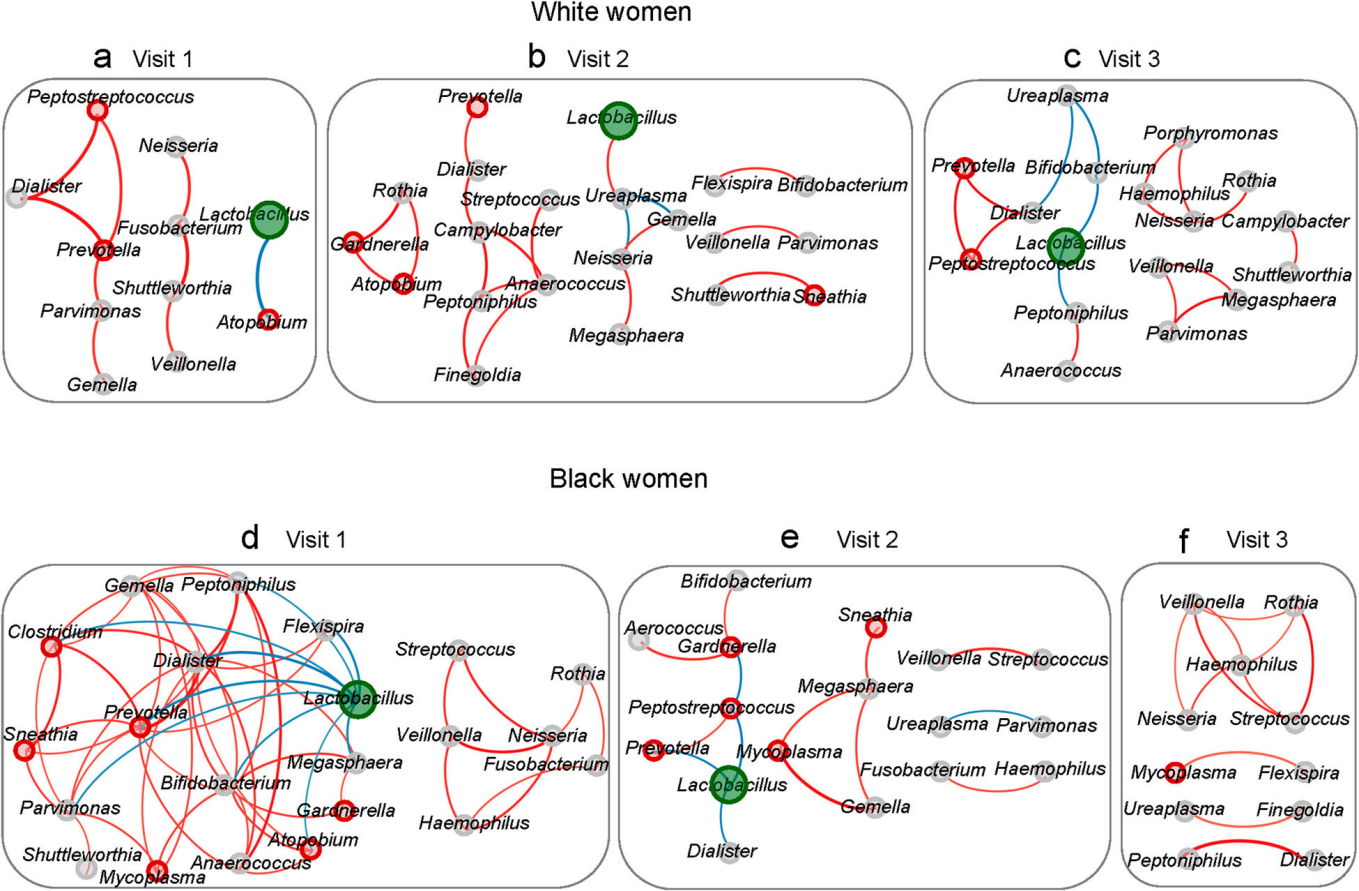
Effect of DMPA on the interactive structure of the vaginal microbiome. Vaginal microbiome from the participants with pre- and post-DMPA administration was analysed. Correlational networks were constructed using pairwise Spearman's rank correlations among genera. In a network system, module represents a set of genera (nodes) linked to each other by many connection lines, while linked by few connections to nodes of other modules. A relationship was considered significant if *r *> 0.7 and *p* < 0.01. Red connections indicate a positive correlation while blue connections a negative correlation. The thickness of a connection line is in proportion to the *r*-value in the correlation. The node for *Lactobacillus* is coloured in green while VNAB in red and NVNAB in grey. The size of a node represents the relative abundance of a genus. The baseline network was defined at visit 1 (a,d), the deviation of the network from the baseline in response to DMPA at visit 2 (b,e), and the long-lasting effect of DMPA on the network at visit 3 (c,f) in Hispanic White (a–c) and Black women (d–f).

At the baseline in White women, 11 genera formed three modules through 9 connections (edges) (Supplementary Table S5) (Figure 4(a)). After DMPA use, the majority (8/11) of the genera reappeared in the networks at both visits 2 and 3. However, except the connection between *Prevotella* and *Dialister*, none of the other connections remained significant at visit 2 or 3 (Figure 4(b–c)). DMPA use markedly increased the numbers of genera (11→21→17) and connections (9→19→16) involved in the networks at visits 2 and 3, while the average node degree (number of connections per genus) remained relatively stable. DMPA did not significantly change the number of VNAB (3→3→2) in the networks (Supplementary Table S5). *Lactobacillus* was present in the baseline visit 1 as well as at visits 2 and 3 in the networks (Figure 4(a–c)).

In Black women, the network was more interactive at visit 1 compared with other visits (Figure 4(d)). There were two large modules composed of 22 genera linked through 56 connections with an average node degree of 2.55. DMPA markedly simplified the network by progressively decreasing the number of genera (22→17→11), the number of connections (56→14→11), and the node degree (2.55→0.82→1.00) in the network (Supplementary Table S5) (Figure 4(e–f)). DMPA also aborted the network associated with *Lactobacillus*. *Lactobacillus* had 9 negative connections at visit 1 but decreased to 3 and 0 connections at visits 2 and 3, respectively. Despite DMPA use, the connection between *Veillonella* and *Streptococcus* was preserved in all 3 visits.

DMPA use did not have a significant effect on the number of VNAB involved in the network at visit 2 but markedly reduced the VNAB in the network at visit 3 in both in White (3→4→1) and Black (6→5→1) women (Supplementary Table S5).

DMPA use reduced the *Firmicutes* to *Bacteroidetes* ratio and *Lactobacillus* to *Prevotella* ratio in Black women.

In the vagina, *Firmicutes* and *Bacteroidetes* were the first and third most abundant phyla in both White and Black women (Supplementary Table S6) (Supplementary Figure 3a). At the baseline, the *F/B* ratios were similar between Black and White women but differed significantly at visit 2 (Supplementary Figure 3b) due to a significant decrease in the *F/B* ratio in Black women (Supplementary Figure 3c). The difference was not significant in visit 3. DMPA treatment did not show a significant effect on the *F/B* ratio at either visit 2 or 3 compared with visit 1 in White women.

**Figure 5.**
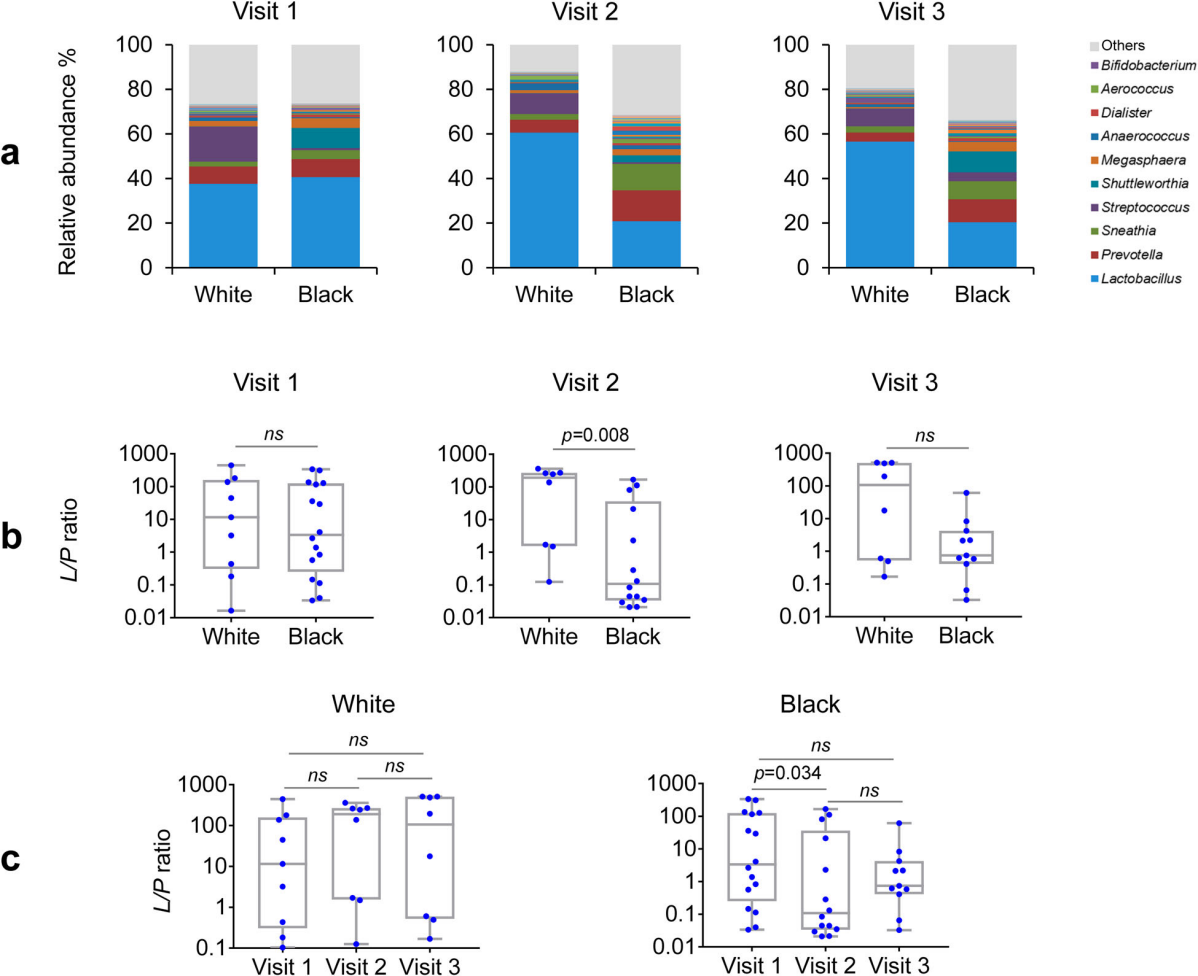
Racial difference in *Lactobacillus* to *Prevotella* ratio in the vaginal microbiome before and after DMPA treatment. Vaginal microbiome from the participants with pre- and post-DMPA administration was analysed. The genus-level community structures are shown for both Hispanic White and Black women at the three visits (a). The “Others” indicated by grey colour is the combined unclassified genera and low abundant genera (<0.1%) that were not included in taxonomic analyses. *Lactobacillus* to *Prevotella* (*L/P*) ratios in the vaginal microbiome after DMPA treatment were compared among and between the three visits in White or Black women and at the three visits (b). The *L/P* ratios were also compared between White or Black women at the three visits (c). *P-*values were calculated using the Kruskal–Wallis test for comparisons among the three visits and the Wilcoxon rank-sum test between two visits or two racial groups. A change in a ratio was considered as significant if *p* < 0.05.

Because *Lactobacillus* and *Prevotella* are the two major genera (Figure 5(a)) and play essential roles in both health and disease in the vagina, they were chosen to represent their phylum-level taxa *Firmicutes* and *Bacteroidetes (F/B)*, respectively. We compared the ratio of *Lactobacillus* to *Prevotella* (*L/P* ratio) between the two racial groups pre- and post-DMPA treatment. The *L/P* ratio essentially mirrored the *F/B* ratio in all comparisons, characterized by similar *L/P* ratios between White and Black women at the baseline. There was a decrease in the *L/P* ratio in Black but not White women at visit 2 compared with visit 1 (Figure 5(b)). At visit 2 but not at other visits, the *L/P* ratio also differed between White and Black women (Figure 5(c)). In contrast, DMPA treatment did not show significant effect on the *L/P* ratio at visit 3 in either White or Black women compared with the baseline visit 1, suggesting that the influence of the vaginal microbiota may be decreased because the concentration of circulating DMPA has been reported to decrease by 3 months post-injection [36].

## Discussion

Our prospective, longitudinal, interventional study indicated a racial difference in the dynamics of the vaginal microbiome in response to DMPA administration. The vaginal microbiome in White women had a transient change and was relative resistant to the effect of DMPA, whereas there was a greater disruption of the bacterial community structure in Black women. These findings highlight the importance of consideration of race in the study of DMPA-related microbiome changes.

In Black women, DMPA use enriched total VNAB and *Prevotella* at visit 2 compared with the baseline visit 1, while no significant effect of DMPA on vaginal microbiome was observed in White women. Several reports studied the effect of DMPA on the vaginal bacterial community but the results are not consistent. In the only study using high throughput 16S rRNA gene survey, Brooks et al*.* reported that DMPA use was associated with lower VNAB prevalence and higher *Lactobacillus iners* [27], while we detected VNAB in all samples analysed regardless of DMPA treatment and an increasing trend of *Lactobacillus* in White women but a decreasing trend of *Lactobacillus* in Black women after DMPA treatment. Brooks’ study differs in several aspects from the present study, which could account for the different findings. Firstly, the previous study compared DMPA users with condom users rather than a baseline measurement from participants prior to the initiation of DMPA treatment. Secondly, the duration of DMPA use was longer than 12 months in most participants with only 11% DMPA users sampled within 30 days when microbiome changes were most prominent as shown in our study, but the analysis was not stratified by the duration. Thirdly, the cross-sectional study by Brook et al*.* compared DMPA users with 78% Black women and other racial groups with condom users with 59% Black women and other groups. Thus, our findings from the present study appear more reflective of DMPA effect because of the women serving their own controls, defined post-exposure time, and separate analyses of Black women from White women. Using qPCR analysis for three *Lactobacillus* species and *Gardnerella vaginalis*, Roxby et al*.* observed a decreasing trend of *G. vaginalis* over a period of one year after DMPA use but did not analyse its abundance at any specific time points [9]. Note that the study by Achilles et al*.* using a qPCR method shows an increasing but not significant trend of *G. vaginalis* at day 30 after DMPA treatment compared with the baseline although its abundance had decreased at day 60 and 180 after DMPA treatment [26]. Using cultivation methods, Mitchell et al*.* found DMPA decreased H_2_O_2_-positive *Lactobacillus* [25] and using tandem-mass spectrometry or phylochips in cross-sectional studies, no significant changes in the vaginal microbiome were detected in DMPA users [29]. The different findings in the latter two studies are not comparable to our study due to different microbial analysis approaches, racial consideration, and statistical methods.

This is the first study demonstrating the effect of DMPA on the interactive bacterial community structure. DMPA use altered the interactive network of the vaginal microbiome in Black women. While the baseline network was large and rich in interactions in Black women, DMPA use markedly simplified the network at visit 2 and almost abolished the entire network at visit 3. Importantly, *Lactobacillus* was the most interactive genus in the baseline network with 9 negative connections but its network was simplified 3 connections at visit 2 and undetectable at visit 3. In contrast, in White women, DMPA use increased both the size and complexity of the network. The positive and negative connections could be reflective of biological cooperative and inhibitive relationships, respectively. Normally, the relationships are believed to be determined mainly by metabolic dependence or exclusion among indigenous bacteria [37] and the complexity of network is in proportion to the diversity of a bacterial community. However, an external force or perturbation such as DMPA might disrupt the dependence. For example, changes in host immune system have been shown to permit colonization of the proximal gut by environmental bacteria in HIV infection [38] and enrich *Flexispira* in experimental SHIV infection [39], highlighting the importance of host-microbial interaction. The opposite changes in the networks in Black women and White women after DMPA treatment might be driven mainly by racial difference in the host-microbial reaction to DMPA rather than by the diversity in the vaginal microbiome. These findings provide another line of evidence supporting the racial difference in the response to DMPA.

Besides separate analyses of the White and Black vaginal microbiomes in response to DMPA treatment, we also compared these two groups at each visit. Ravel et al. classify the vaginal microbiome into five CSTs based on the abundance of *Lactobacillus* and *Gardnerella* [11]. CST types I, II, III, and V are dominated by one or more species of *Lactobacillus*, while type IV is highly diverse. The CSTs dominated by species of *Lactobacillus* (groups I, II, III, and V) are found in 80.2% and 89.7% of Asian and White women, respectively, but in only 59.6% and 61.9% of Hispanic and Black women, respectively. In our cohort, at the baseline, *Lactobacillus* was the most abundant genus but its median relative abundance was only 29.02% in White and 34.88% in Black women, while the relative abundance of *Gardnerella* was below 0.3% in both groups (Table S5). These profiles indicate that the two vaginal microbiomes were globally similar to each other resembling the type IV CST despite of the observed difference in unweighted β-diversity and the more abundant *Shuttleworthia*, *Finegoldia*, and *Clostridium* in Black than White women. DMPA use markedly diverted the two microbiomes in the following aspects. (i) *Lactobacillus* became more abundant in White than Black women, essentially having converted the White microbiome to a *Lactobacillus-*dominated CST (median abundance 70.87% at visit 2 and 80.73% at visit 3) and markedly depleted *Lactobacillus* in the Black microbiome (2.13% in visit 2 and 16.32% in visit 3). (ii) While total VNAB was 4.73 fold more abundant in the Black than White microbiome at the baseline, the difference was markedly widened to 24.08 fold at visit 2 and partially recovered to 6.16 fold at visit 2. In particular, VNAB genera *Prevotella, Sneathia,* and *Clostridium* were more abundant in the Black than the White microbiome after DMPA treatment. (iii) After DMPA use, the microbiome of Black women became more diversified than that of the White women as indicated by α-diversity at visits 2 and 3. (iv) The β-diversity of microbiomes differed between White and Black women weighted as measured by UniFrac distance at visits 2 and 3. These findings suggest that the differential response to DMPA is more likely due to the host difference in race rather than pre-existing microbiome differences between White and Black women.

Several changes induced by DMPA in the Black vaginal microbiome may potentially enhance HIV acquisition as similar vaginal microbiome profiles have been associated with high risk of HIV acquisition. (i) *Lactobacillus* deficiency: *Lactobacillus* inhibits HIV-1 replication [40] and its deficiency has been linked to high HIV-1 acquisition in sex workers in Kenya [19] and healthy South African women [41]. (ii) VNAB: BV is a polybacterial, proinflammatory state that increase susceptibility to HIV infection by mucosal damage and promotion of viral expression and propagation [42–44]. BV have been consistently associated with an increased risk of HIV infection in Africa and around the world [21,22]. In healthy women, VNAB are associated with increased inflammation and HIV acquisition [41]. (iii) high-diversity vaginal microbiome: CST IV has been shown to be more prevalent among women who acquire HIV-1 than other CSTs [41]. Thus, alteration of vaginal microbiome might be one of the mechanisms of DMPA-induced high risk of HIV acquisition.

The main strength of this study is its prospective nature. Because of this study design, we were able to collect samples from participants prior to initiation of DMPA treatment. Thus, the microbiome differences observed in the same subjects are more likely to be due to DMPA use rather than confounding factors present in case or control groups of unrelated individuals. The second strength is the stratification by racial status of the participants, which otherwise masks the effect of DMPA. The third strength is the application of the comprehensive network analysis to allow us to detect the drastic modification of the interactive structure in the vaginal microbiome in Black women after DMPA treatment. The fourth strength is the application of FDR in the multiple comparisons to avoid erroneously reported something as significant that was due to chance.

The limitations pertinent to this study are related to the small sample size and the exploratory nature. Our analytic strategy was to discover major differences given the susceptibility of a small-scale study to false discoveries. We focus on taxa at higher phylogenetic ranks because the major taxa are stable and reproducible while species-level taxa are subject to inaccuracy due to sequencing errors, inadequate classification related to short 16S rRNA gene reads, and fluctuation in abundance pertinent to low abundant taxa. Despite the small sample size, it was sufficient to allow detection of the main differences with statistical difference, our study was subject to type-II errors (i.e. failing to detect an effect that is present in a small-scale study) as indicated by fact that many if not most of the differences examined were not significant after adjustment for multiple testing. Thus, our results should be corroborated on a larger group of subjects.

In conclusion, the vaginal microbiome exhibited a racial difference in the DMPA-mediated vaginal microbiome changes. The vaginal microbiome is relatively resistant to DMPA in Hispanic White women but sensitive in Black women. In Black women, DMPA use not only changed the taxon membership and abundance but also damaged the interactive network in the vaginal microbiome. The pathophysiological nature of the microbiome changes following DMPA use, albeit not determined in the present study, are likely to be harmful given the enrichment of the total VNAB and *Prevotella*, disappearance of the beneficial bacterial *Lactobacillus* network, and decrease of the *F/B* and *L/P* ratios. These DMPA-induced microbial changes might contribute to an increased risk of HIV-acquisition in Black women. Although the DMPA-mediated taxon changes appeared to be transient, the incomplete recovery of the microbiome and disappearing interactive network 3 months after the exposure suggest a potential long-lasting effect. A better understanding of how DMPA alters vaginal microbiome in women with diverse backgrounds may help develop an effective strategy for HIV prevention.

## Materials and methods

### Study design, patient recruitment, and sample collection

This was a prospective, longitudinal, interventional study to evaluate the impact of DMPA on the vaginal microbiome. Participants were women receiving healthcare at Rutgers, New Jersey Medical School clinics located in Newark, NJ. Women who desired to use Depo-Provera, met the primary screening criteria as described below, and were willing to sign the informed consent form were recruited and compensated, as previously described [7]. The inclusion criteria were: (i) non-pregnant women aged 18–35 years who visited the clinic; (ii) medically eligible to use DMPA and having no history of sexually transmitted infections including HIV, syphilis, chlamydia, and gonorrhea; (iii) no use of hormonal contraception in the previous 2 months; (iv) no sexual intercourse for 3 days prior to the clinical enrolment visits and no measurable PSA (prostate-specific antigen, a surrogate marker for sperms during sexual intercourse) by ELISA (PSA/KLK3 Human ELISA Kit, Thermo Fisher Scientific, Waltham, MA USA) at visits 2 and 3 [45–47]; and (v) no use of any antibiotics before samples were collected.

Participants received DMPA with a single dose of 150 mg at the time of enrolment. A mid-vaginal swab (Isohelix DNA Buccal Swabs, Boca Scientific) was acquired as a baseline sample before DMPA administration (visit 1), 1 month (visit 2) and 3 months after (visit 3) DMPA injection, by one clinician.

This study was approved by two institutional review boards (IRB), namely Rutgers University Biomedical and Health Sciences IRB (protocol number: Pro20140000011) for the patient recruitment part of the study and New York University School of Medicine IRB (protocol number: i12-03834) for the microbiome component of the study. Races were self-identified by the participants. Participants were self-reported as either Black or Hispanic White, with none as American Indian and Alaska Native, Asian, or Native Hawaiian and Other Pacific Islander.

### DNA extraction and 16S rRNA gene library preparation

Total genomic DNA was extracted from the swab specimens using a DNeasy PowerLyzer PowerSoil DNA extraction method (QIAGEN, Germantown, MD), as previously described [39]. From the extracted DNA, the V3 and V4 regions of bacterial 16S rRNA genes were amplified using the primer set 347F 5′-GGAGGCAGCAGTRRGGAAT and 803R 5′-CTACCRGGGTATCTAATCC, which we previously designed [30]. PCR reactions were carried out as previously described [48]. PCR products were purified using Agencourt AMPure XP (Beckman Coulter Life Sciences, IN) and quantified using Qubit 2.0 Fluorometer (thermo fisher scientific, NJ). Amplicon libraries were pooled at equimolar concentrations and sequenced on Illumina MiSeq platform. Three types of negative controls were included to detect exogenous contamination: (i) Template-negative controls (PCR reaction without DNA template) along with the samples in PCR reactions; (ii) Specimen-negative controls (use PBS in lieu of a human specimen) along with the samples while extracting DNA from the human samples. (iii) treatment-negative controls (visit 1 before DMPA as baseline) that were run under the same PCR and sequencing condition as the samples from same patients.

### Taxonomic classification

Using the QIIME pipeline 1.9.1 [49], reads were demultiplexed and filtered using default parameters with quality score ≤20. The sequences were grouped into operational taxonomic units (OTUs) and classified to taxonomic levels from the phylum to genus levels. Chimeric OTUs were excluded by ChimeraSlayer. OTUs that were unclassifiable to specific taxa at these levels were excluded from further analysis.

### α- and β-diversity measurement

α-diversity was calculated by Monte Carlo permutations using compare_alpha_diversity.py, a built-in function in the QIIME pipeline, which included observed taxa for total species richness, Chao1 predicted taxa estimated for total species richness, and Shannon diversity index for both richness and evenness indices. Variation in alpha-diversity measures was tested using Kruskal–Wallis test for comparisons among the three clinical visits and using Wilcoxon signed-rank for paired samples between two visits or Wilcoxon rank-sum test for unpaired samples between two racial groups. β-diversity was analysed using weighted and unweighted UniFrac distance matrices by principal coordinate analysis (PCoA) [50] and statistically tested using microbiome regression-based kernel association test (MiKART) [51].

### Taxonomic analysis of differences before and after DMPA treatment

Comparisons among and between the clinical visits before and after DMPA were mainly performed at the genus level. To reduce noise and false discoveries, genera with very low abundance (<0.1%) were excluded from the comparisons. Comparison between any two of the three visits was performed using Wilcoxon signed-rank for paired samples. Comparison between Black and White women performed using Wilcoxon rank-sum test for unpaired samples. The statistical tests were two-sided, with a *p*-value < 0.05 considered of nominal statistical significance. A false discovery rate (FDR)-adjusted *p*-value (*q-*value) < 0.20 was used as the threshold for significance [52–55]. All statistical tests were conducted using R version 3.2.1.

### Correlational network analysis

Networks among bacterial genera were determined by correlation analysis [56]. Appearance or disappearance of correlation between pairs of bacterial genera at difference time points and racial groups were described as they suggest relationships potentially important in the bacterial community structure and interactions [57,58]. First, correlation matrixes were measured by calculating the pairwise Spearman's rank correlations between all genera whose average relative abundance was higher than 0.1% across all samples correspondingly. To minimize false discoveries, a relationship was considered significant only when the absolute *r-*value > 0.7 and *p *< 0.01. We defined the baseline network at visit 1, quantified the deviation of the network from the baseline in response to DMPA at visit 2, and described the long-lasting effect of DMPA on the network at visit 3. Furthermore, we evaluated the impact of DMPA on VNAB involved in the network. Correlation analyses and matrix were generated in R environment by corrplot [59], igraph [60] and Hmisc [61] packages. Visualization of the network was performed in the platform of Gephi [62].
